# Corrugated Textile based Triboelectric Generator for Wearable Energy Harvesting

**DOI:** 10.1038/srep45583

**Published:** 2017-03-28

**Authors:** A Young Choi, Chang Jun Lee, Jiwon Park, Dogyun Kim, Youn Tae Kim

**Affiliations:** 1 IT Fusion Technology Research Center and Department of IT Fusion Technology, Chosun University, Gwangju 61452, Korea

## Abstract

Triboelectric energy harvesting has been applied to various fields, from large-scale power generation to small electronics. Triboelectric energy is generated when certain materials come into frictional contact, e.g., static electricity from rubbing a shoe on a carpet. In particular, textile-based triboelectric energy-harvesting technologies are one of the most promising approaches because they are not only flexible, light, and comfortable but also wearable. Most previous textile-based triboelectric generators (TEGs) generate energy by vertically pressing and rubbing something. However, we propose a corrugated textile-based triboelectric generator (CT-TEG) that can generate energy by stretching. Moreover, the CT-TEG is sewn into a corrugated structure that contains an effective air gap without additional spacers. The resulting CT-TEG can generate considerable energy from various deformations, not only by pressing and rubbing but also by stretching. The maximum output performances of the CT-TEG can reach up to 28.13 V and 2.71 μA with stretching and releasing motions. Additionally, we demonstrate the generation of sufficient energy from various activities of a human body to power about 54 LEDs. These results demonstrate the potential application of CT-TEGs for self-powered systems.

Energy-harvesting technologies obtain electric energy from the ambient environment, e.g., solar^1^, wind^2^, thermal^3^, and mechanical energy^4^,^5^. Energy harvesting has emerged as one of the most promising solutions for the rapidly increasing energy crises and global warming issues. Wearable energy-harvesting technologies are attractive for reducing the size and weight of electronic devices. Wearable energy sources should be flexible, lightweight, and ease to use. Wearable devices typically use conventional electrochemical batteries as energy sources. However, a battery does not provide energy consistently because it has a limited lifetime and is not durable. Recently, simple, highly efficient, and cost-effective triboelectric generators (TEGs) have been developed^6^,^7^,^8^,^9^,^10^,^11^,^12^,^13^,^14^,^15^,^16^,^17^,^18^. Among them, fiber/textile-type TEG^19^,^20^,^21^,^22^,^23^,^24^,^25^,^26^,^27^,^28^ technologies provide an energy source for self-powered devices, e.g., wearable devices, electronic skin, bio-medical devices, and portable electronics, because of their lightweight, flexibility, and comfort. In principle, triboelectric energy-harvesting technologies operate by contact electrification and electrostatic induction^6^. As our body movements, e.g., stretching, pressing, and rubbing, are frequent and easily accessible mechanical-energy sources, textile-based TEGs integrated into clothing can effectively convert this mechanical energy into electrical energy. Most previously developed textile-based TEGs have generated energy based on pressing and rubbing motions^19^,^20^,^21^,^22^,^23^.

In this study, we propose a corrugated textile-based triboelectric generator (CT-TEG), which generates energy not only by pressing and rubbing but also by stretching. The CT-TEG is sewn into a corrugated structure using the textile characteristics. As a result, the output voltage of the triboelectric generator 28.13 V, 119.1 V, 11.2 V at stretching, pressing and rubbing motions, respectively. Also, the CT-TEG generated energy under mechanical deformation of wrist, arm and foot and light up 54 light emitting diodes connected in series. The proposed CT-TEG is an extremely promising power supply for wearable devices.

## Results

### Structure of corrugated textile-based triboelectric generator

**Figure 1:**
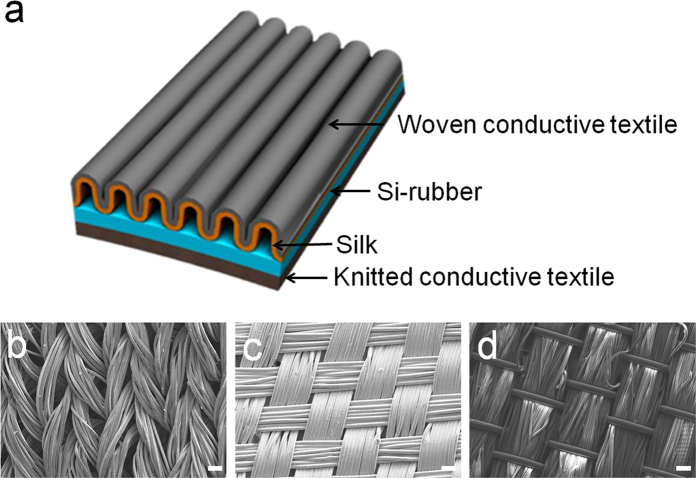
Corrugated textile-based TEG structure and morphology. (**a**) Schematic illustration of the CT-TEG. SEM images of the (**b**) woven conductive textile, (**c**) knitted conductive textile and (**d**) silk. (scale bar: 1 mm).

Figure 1(a) shows the design of the corrugated textile-based triboelectric generator (CT-TEG). The CT-TEG consists of the following two layers: silk with a woven conductive textile on the top and Si-rubber with a knitted conductive textile on the bottom. The SEM images of woven conductive textile and knitted conductive textile shown in Fig. 1(b,c), respectively. The top layer is composed of a corrugated structure that is sewn to the bottom layer (Supplementary Fig. S1). Such a corrugated structure enhances the level of effective strain applied in the silk layer and drives the shape of the generator to the original state upon release of the applied force. The corrugated structure can achieve reversible contact (stretched) and non-contact (released) states, as demonstrated in Supplementary Fig. S2. The CT-TEG can be stretched up to 120%. Figure 1(d) is the SEM image of the silk textile as the top layer. The surface of the silk textile has roughness, which leads to efficient friction. The average diameters of the silk textile were 16 μm.

### Working mechanism of corrugated textile-based triboelectric generator

**Figure 2:**
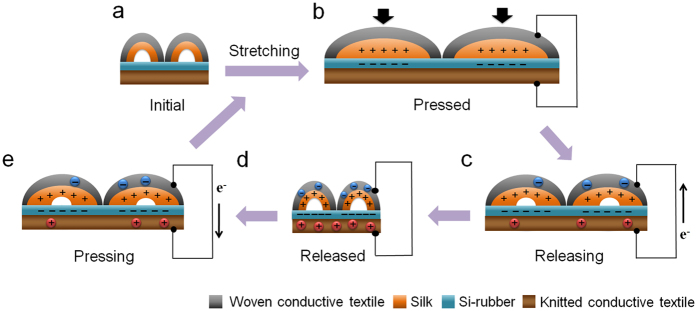
Schematic illustration of the suggested working principle of the CT-TEG. (**a**) Initial state without mechanical force. (**b**) Triboelectric charge distribution at full-contact state and (**c**) releasing state. (**d**) Triboelectric potential at full-separation state and (**e**) pressing state.

The power-generation mechanism of the CT-TEG is illustrated in Fig. 2. The CT-TEG enables energy generation by a conjunction of triboelectrification and electrostatic induction. The silk and Si-rubber are used as positive and negative triboelectric active materials. Since the silk and Si-rubber have different electron-attracting abilities, a surface charge is transferred when the two are brought into contact. In the initial state, without any motion, no charge is transferred (Fig. 2(a)). Once the CT-TEG is stretched, the TEG brings the silk and Si-rubber into full contact (Fig. 2(b)). Any motion will lead to a contact or separation between the silk and Si-rubber. The silk is charged positively and the Si-rubber is charged negatively because of their triboelectric characteristics.

When the two triboelectric materials are brought into contact, electrons move from the silk to Si-rubber because Si-rubber has a higher surface-electron affinity than silk. Consequently, in the CT-TEG, there is a net negative charge on the Si-rubber and a net positive charge on the silk^26^. Removing the external force causes a separation. Electrons flow from the Si-rubber-coated conductive textile electrode to the silk/conductive textile electrode (Fig. 2(c)). When the silk and Si-rubber are separated by the maximum distance, an electrical equilibrium is formed (Fig. 2(d)). When external force is applied to bring the silk and Si-rubber into contact, it causes electrons to flow from the silk/conductive textile electrode to the Si-rubber-coated conductive textile electrode (Fig. 2(e)). With the stretching, pressing, and rubbing motions, the surfaces of the silk and Si-rubber come into contact and rub against each other; thus, triboelectric charges are generated and distributed over the surface.

### Electrical performance of corrugated textile-based triboelectric generator

**Figure 3:**
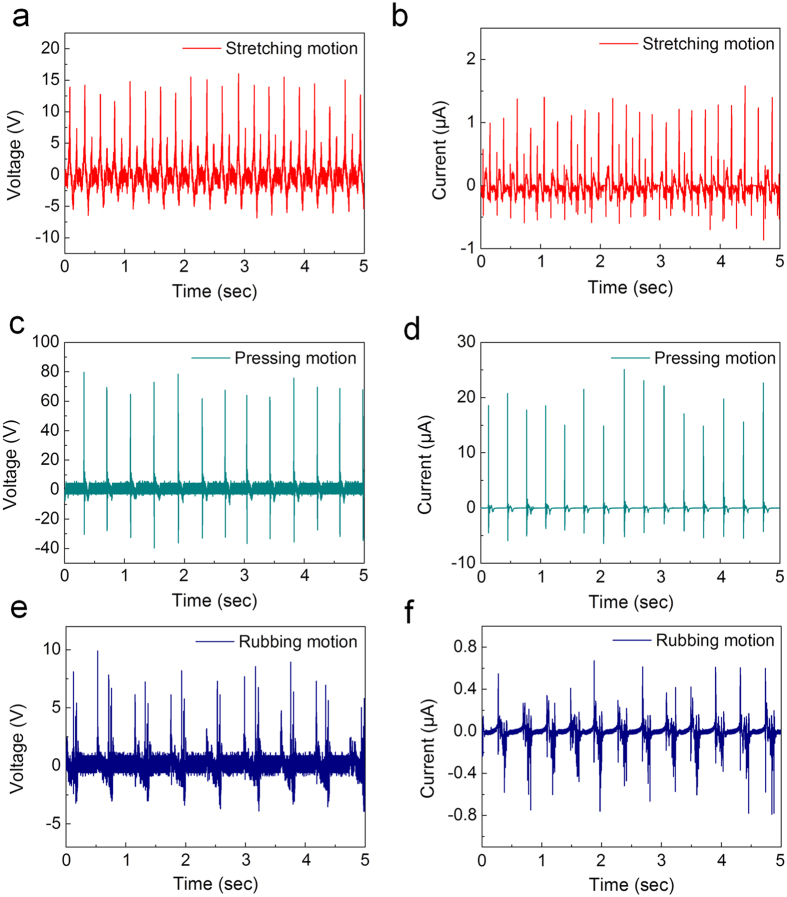
Open-circuit voltage and short-circuit current of the CT-TEG. (**a**,**b**) The output performances under stretching state, (**c**,**d**) pressing state and (**e**,**f**) rubbing state.

Open-circuit voltage and short-circuit current were measured for the CT-TEG under stretching, pressing and rubbing states. In stretching and releasing motions, the CT-TEG produced an open-circuit voltage of 28.13 V and a short-circuit current of 2.71 μA, as shown in Fig. 3(a,b), respectively. To investigate the effect of stretching frequency, it was varied from 2 to 4 Hz (Supplementary Fig. S3). It was evident that the voltages increased gradually with stretching frequency. At each frequency, the voltage values were obtained as follows: 7.45 V (2 Hz), 10.26 V (3 Hz), and 22.45 V (4 Hz). Under a vertical pressing force of 1 kgf, the CT-TEG exhibited a repeatable and consistent voltage of 119.1 V and a current of 26.1 μA (Fig. 3(c,d)). Additionally, the output voltage and current increased with the external pressing force. (Supplementary Fig. S4) Under a pressing force of 0.2 kgf to 0.6 kgf, the voltage increased from 23.9 V to 60.5 V. This can be because more friction and deformation of silk and Si-rubber under higher external mechanical force increases the amount of generated triboelectric charges. The CT-TEG sliding mode operates at a frequency of 2 Hz; the electrical output shows 11.2 V and 0.84 μA (Fig. 3(e,f)). Thus, the corrugated structure of the CT-TEG generates power regardless of the motion.

**Figure 4:**
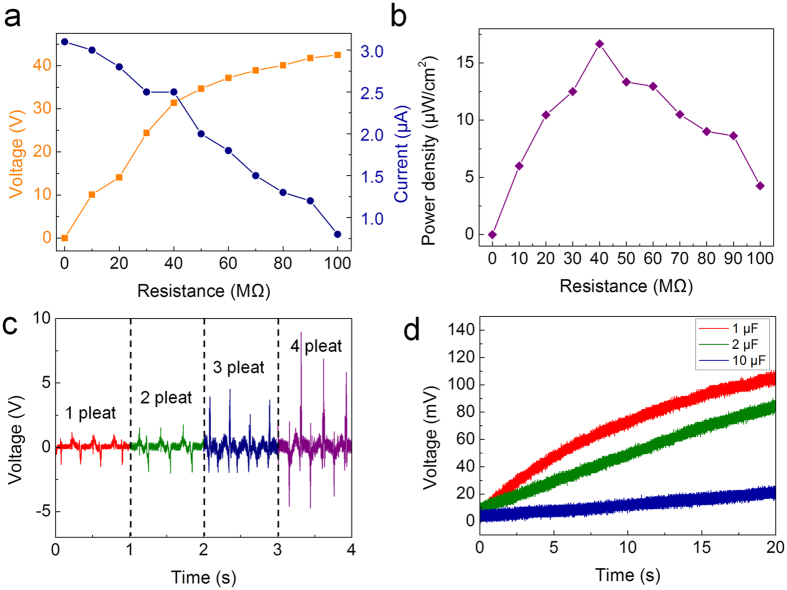
Output results of the CT-TEG for stretching motion. (**a**) Output voltage and current with different resistance values. (**b**) Output power density with external resistance loads. (**c**) Output vs. corrugated density. (**d**) Charging voltage and time of the capacitors.

Figure 4 shows the output performance of the CT-TEG for stretching motion. The maximum output voltage increased with load resistance, whereas the maximum voltage decreased (Fig. 4(a)). Accordingly, the instantaneous output power calculated using W = I^2^ peak × R was 16.6 μW/cm^2^ for a load resistance of 40 Ω, as shown Fig. 4(b). Figure 4(c) shows the output results vs. corrugated density. It is observed that the triboelectric output voltage increases with the corrugated density. Typically, a larger contact area produces more triboelectricity^13^. The charging of capacitors with different capacitance values has been studied experimentally, as shown in Fig. 4(d). It was observed that in the CT-TEG, the 1 μF capacitor charged to 114 mV in 20 s under periodic stretching and releasing. Additionally, the 2 μF capacitor charged to 99 mV. Therefore, the proposed CT-TEG is sufficient as an energy harvesting unit for a power source that employs stretching and releasing motions.

**Figure 5:**
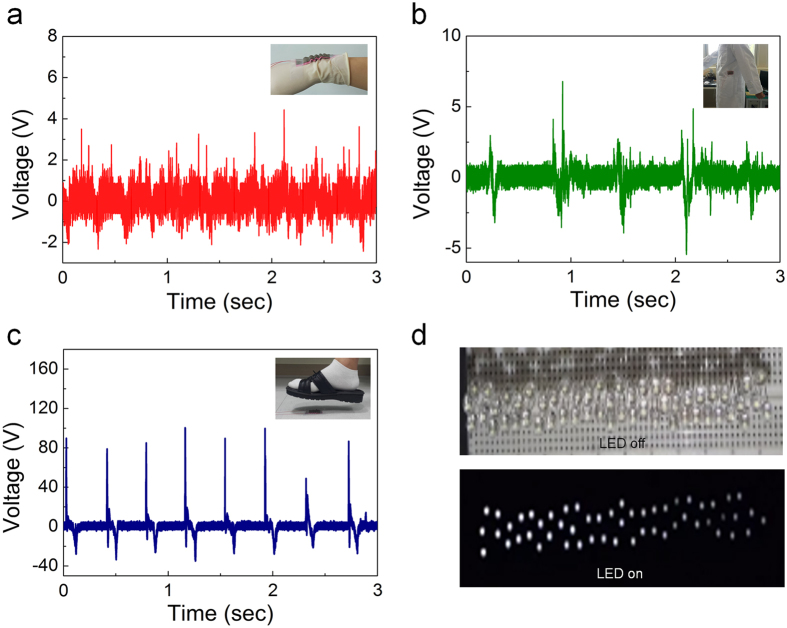
Applications of CT-TEG. (**a**) Output voltage when bending and releasing the wrist. (**b**) Output voltage at the side of the body, harvesting energy from the arm rubbing. (**c**) Output voltage harvesting energy from footsteps. (**d**) LED before and after lighting up.

To investigate the capability of the CT-TEG as a power source, we considered different parts of the human body. Here, the movement of the wrist, knee, and foot are the most important mechanical-energy sources supplied by the human body. As shown in Fig. 5(a), a stretchable textile-based CT-TEG is placed over the wrist joint. The CT-TEG stretches when the wrist bends and relaxes when the wrist straightens, generating output voltage signals. The triboelectric voltage generated by the wrist joints is about 6.8 V. Figure 5(b) shows a corrugated textile-based CT-TEG attached to the side of the body to harvest energy from the arm rubbing. The output voltage is about 8.1 V. Figure 5(c) shows the corrugated textile-based CT-TEG generating energy from footsteps. Its output voltage is about 110.1 V, which is capable of lighting up about 54 light-emitting diodes (LEDs) (Fig. 5(d)). Therefore, the proposed corrugated structure of the textile-based TEG has several advantages, e.g., harvesting different kinds of mechanical energy from the human body. It effectively converts ambient mechanical energy into electricity, which can be used to drive small electronics such as wearable devices.

## Discussion

In this paper, we propose a new corrugated structure for a textile-based triboelectric generator that can be attached to the surface of the human body to harvest mechanical energy. The fabric of the presented triboelectric generator is sewn into a corrugated structure that achieves an effective air gap without additional spacers. The maximum output voltages of the triboelectric generator are 28.13 V, 119.1 V and 11.2 V for stretching, pressing, and rubbing motions, respectively. Additionally, we demonstrated the generation of sufficient energy from various activities of the human body to operate 54 LED bulbs. It is expected that the stretchable and high-performing corrugated textile-based TEG can be widely used for wearable energy-harvesting systems.

## Methods

### Fabrication of the CT-TEG

A commercially available knitted conductive textile (Ag-plated nylon, DEV-10070) and a woven conductive textile (Sn/Cu/Ag-plated nylon, DEV-10056) were used for fabricating the CT-TEG. The top layer consisted of silk with the woven conductive textile attached through backstitch. The production of Si-rubber involves mixing the EcoFlex0050 solution in a 1:1 ratio for 15 min using a glass rod. The bottom layer is composed of Si-rubber coated on the knitted conductive textile substrate, which is kept at room temperature for 3 h. The top layer is composed of a corrugated stretchable textile sewn onto the bottom layer. Narrow air gaps were created in the CT-TEG between the top and bottom layers. The size of CT-TEG is 5 × 3 cm^2^.

### Characterization and measurement of the CT-TEG

The morphologies of the silk textile were examined through SEM(JSM 840-A, JEOL. Ltd, Japan). A pushing tester (JIPT-100, JUNIL Tech. Ltd, Korea) was used to apply forces to the CT-TEG. The triboelectric performance was measured using an oscilloscope (MSO9104A, Agilent Tech., USA) and SMU (B2911A, Agilent Tech., USA).

## Additional Information

**How to cite this article**: Choi, A. Y. *et al*. Corrugated Textile based Triboelectric Generator for Wearable Energy Harvesting. *Sci. Rep.* **7**, 45583; doi: 10.1038/srep45583 (2017).

**Publisher's note:** Springer Nature remains neutral with regard to jurisdictional claims in published maps and institutional affiliations.

## Supplementary Material

Supplementary Information
