# Supplementary material for: Corrugated Textile based Triboelectric Generator for Wearable Energy Harvesting
Source: Sci Rep. 2017 Mar 28;7:45583. doi: 10.1038/srep45583 (PMC5368658; doi:10.1038/srep45583)
Supplement: Supplementary Information [file srep45583-s1.pdf]

SUPPLEMENTARY INFORMATION

Corrugated Textile based Triboelectric Generator for Wearable Energy Harvesting

A Young Choi, Chang Jun Lee, Jiwon Park, Dogyun Kim and Youn Tae Kim*

IT Fusion Technology Research Center and Department of IT Fusion Technology, Chosun University, Gwangju 61452, Korea

[*] E-mail: petruskim@chosun.ac.kr

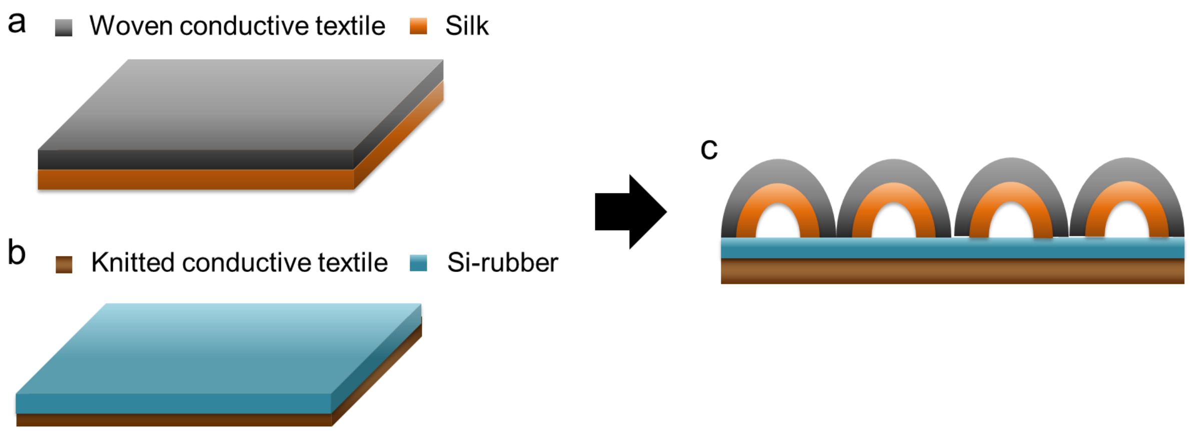

Figure S1 | Configuration of the CT-TEG. (a) The top layer consists of silk with a woven conductive textile electrode; (b) The bottom layer consists of Si-rubber with a knitted conductive textile electrode; (c) Fabricated CT-TEG with top and bottom layers.

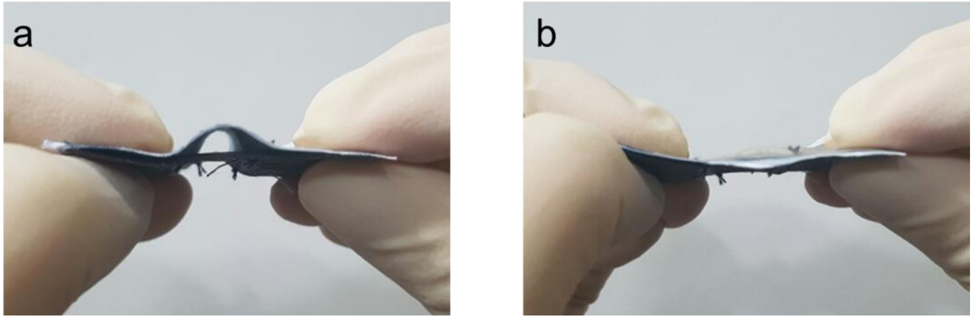

Figure S2 | Photographs of the CT-TEG at (a) released and (b) stretched states.

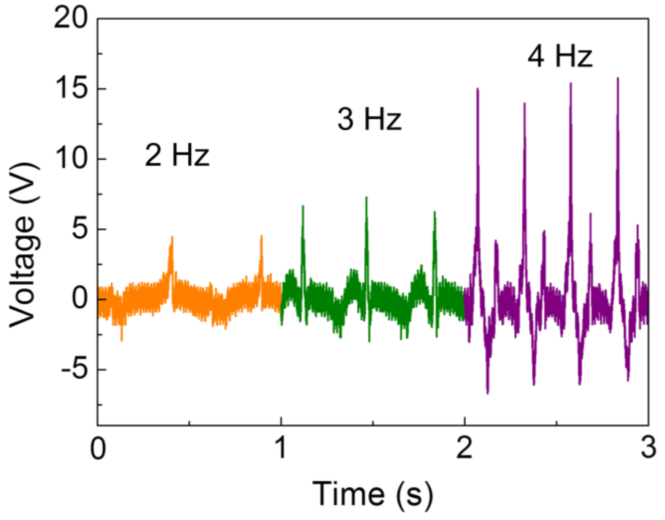

Figure S3| Output voltage for different stretching frequencies.

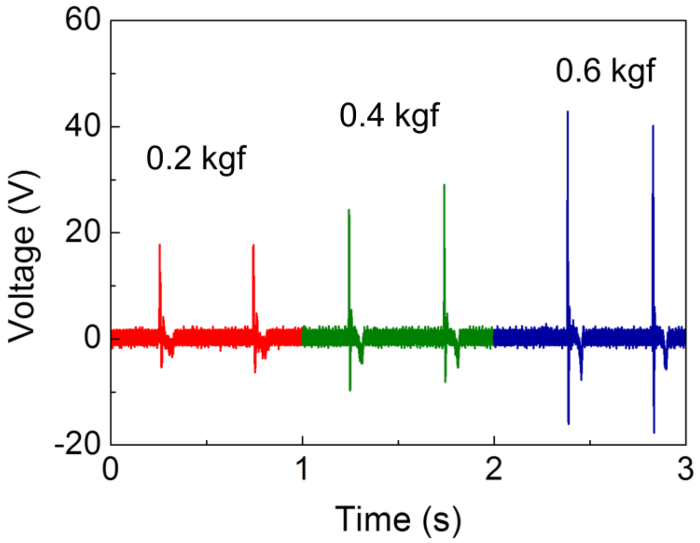

Figure S4| Output voltage for external pressing forces for stretching motion.
